# Supplementary material for: The Effect of Exogenous Nitrate on LCO Signalling, Cytokinin Accumulation, and Nodule Initiation in Medicago truncatula
Source: Genes (Basel). 2021 Jun 28;12(7):988. doi: 10.3390/genes12070988 (PMC8305252; doi:10.3390/genes12070988)
Supplement: Supplementary file 1 [file genes-12-00988-s001.zip › genes-1242154-supplementary.pdf]

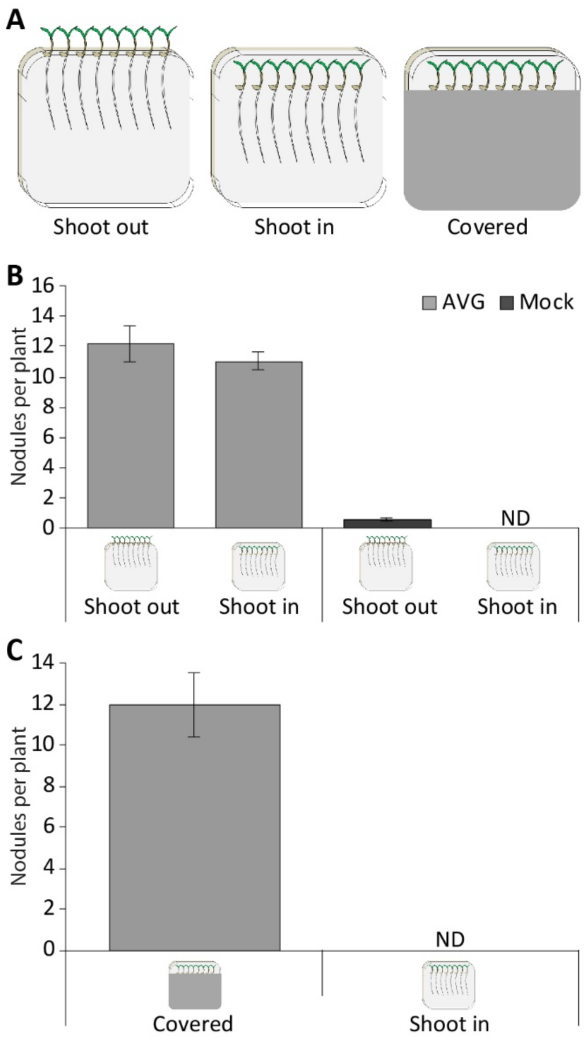

Supplemental Figure S1. Setting up an AVG-independent plate system for *Medicago truncatula* nodulation. **(A)** Schematic representation of three tested inoculation systems; “Shoots out” of the plate, “Shoots in” the plate and shoots in the plate with roots “Covered” by aluminum foil. **(B)** Effect of the “Shoots out” and “Shoots in” system on nodulation on plates. AVG used as a positive control. **(C)** Effect of root covering on nodulation on plates **(B-C)** 14 Days Past Inoculation, bars represent means + SE (n=40).

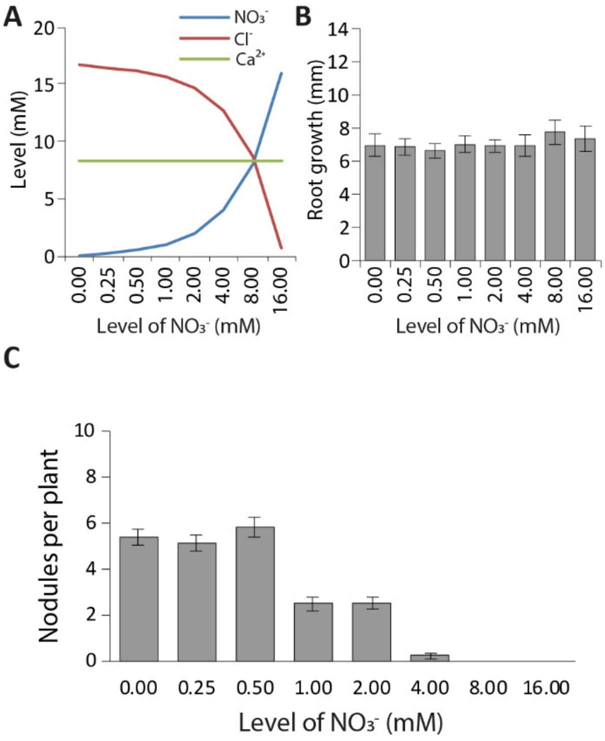

Supplemental Figure S2: Nitrate inhibition of nodulation on Fåhraeus plates. **(A)** Schematic representation of the increase in nitrate levels. **(B)** Effect of increasing levels of nitrate on primary root growth 5 DPG (days post Germination). **(C)** Effect of nitrate on nodulation on plates at 14 DPI. Bars represent means $\pm$ SE (n=40).

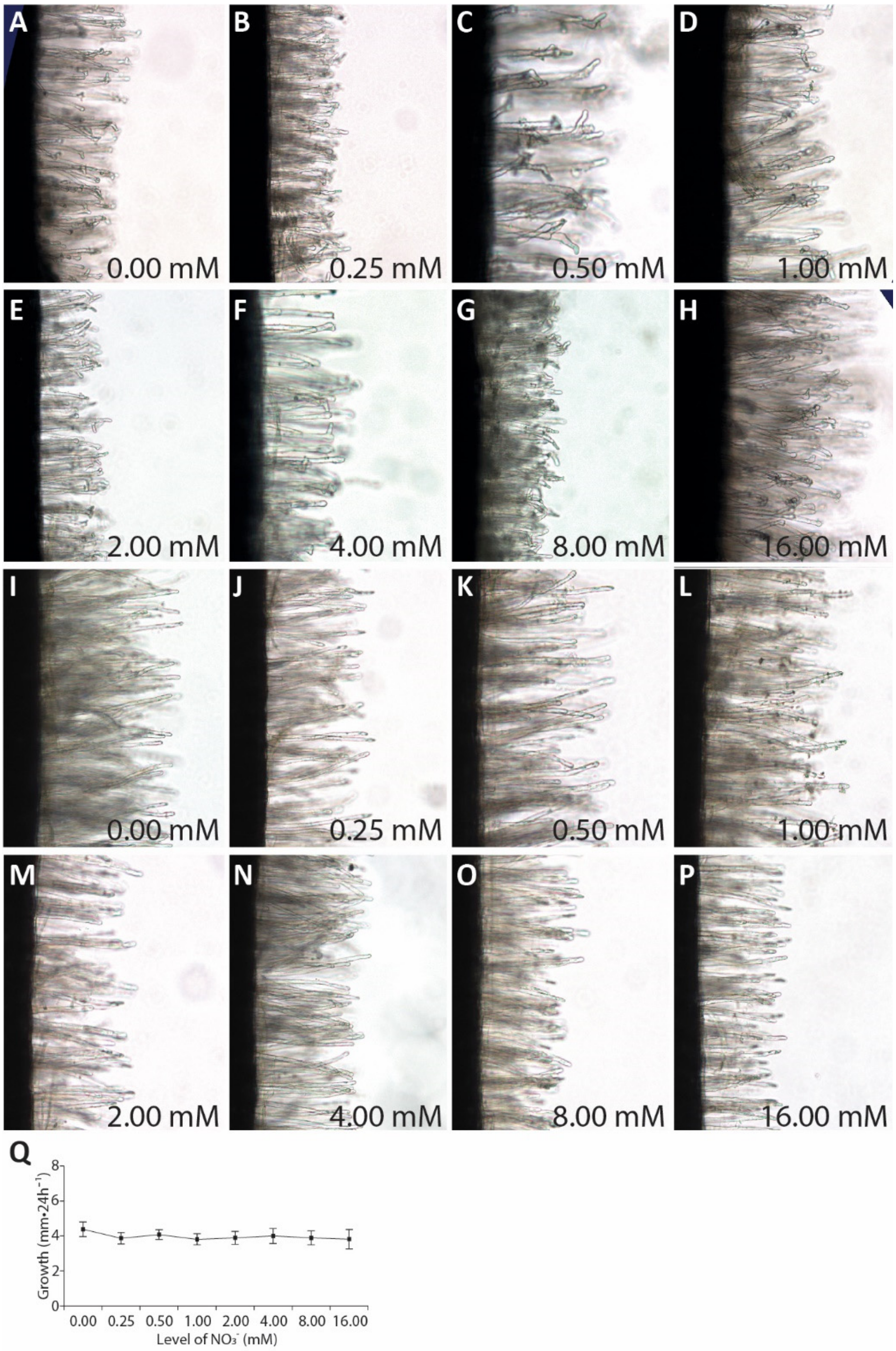

Supplemental Figure S3. Root hair deformation is not affected by elevated levels of nitrate. **(A-H)** LCO-induced root hair deformation at increasing levels of nitrate **(I-P)** Mock-treated roots at increasing levels of nitrate, these pictures are representative of 10 biological replicates divided over two independent experiments. **(Q)** the effect of increased nitrate on primary root growth 2 DAG (days after germination). Bars represent means ± SE (n=10).

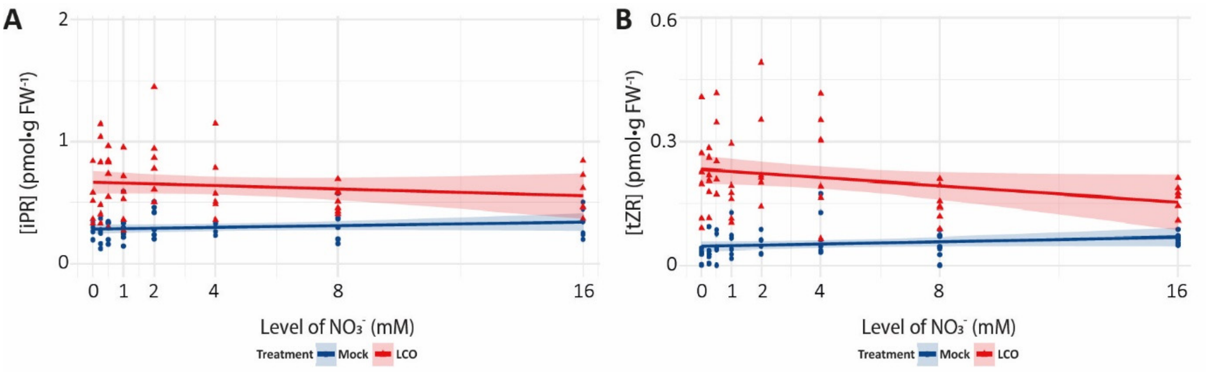

Supplemental Figure S4: The effect of increasing levels of nitrate on iPR and tZR accumulation in the Medicago root susceptible zone. Concentrations of **(A)** iPR, **(B)** tZR, were measured per gram fresh weight using UPLC-MS/MS (n=6) in samples taken from the root susceptible zone and treated with mock or LCO for 3 h.

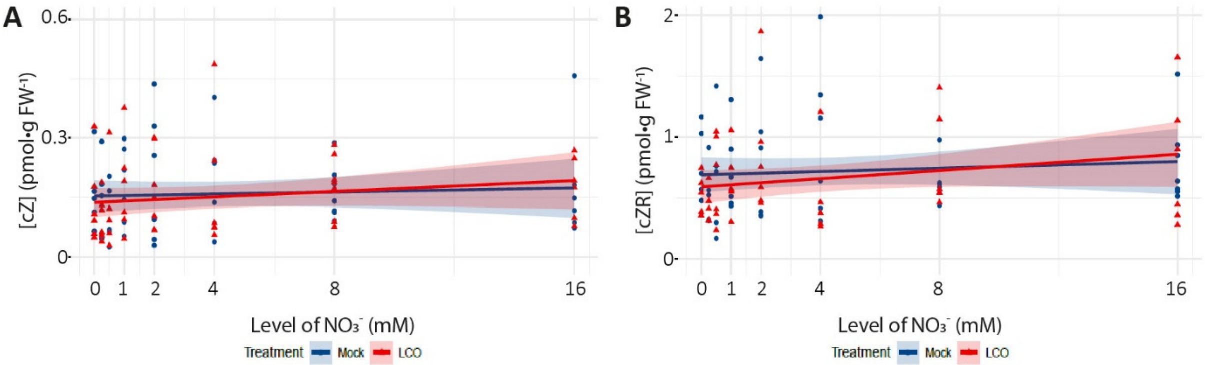

Supplemental Figure S5: The effect of increasing levels of nitrate on cZ and cZR accumulation in the Medicago root susceptible zone. Concentrations of **(A)** cZ, **(B)** cZR, were measured per gram fresh weight using UPLC-MS/MS (n=6,,) in samples taken from the root susceptible zone and treated with mock or LCO for 3 h.

Supplemental Table S1. Sequences and primers used in this study (*M. truncatula* genome v4.01)

Primer name	Sequence	Gene ID
MtACD_Fw	AAACAAAGTGCGGAAATTGG	Medtr8g107670
MtACD_Rv	GGGATCTTGGTCAACGAGAA	
MtNIN_Fw	GGGAGAAAGTCCGGGGACAA	Medtr5g099060
MtNIN_Rv	GACACACACCGATGCTCTTTGC	
MtACT_Fw	GCAAAGGCAGAATATGATGAAT	Medtr2g008050
MtACT_Rv	CCACTATGACTGCCAGAACACTTA	
MtPTB_Fw	TGAACCAGTGCCTGGAATCCT	Medtr3g090960
MtPTB_Rv	CGCCTTGTCAGCATTGATGTC	
MtUBQ_Fw	CACCTCCAATGTAATGGTCTTTCC	Medtr4g091580
MtUBQ_Rv	CCCTTCATCTTGTCCTTCGTCTG	

Supplemental Table S2. Multiple reactions monitoring (MRM) transitions table. Transitions for all cytokinins and corresponding internal standards used in this study.

Number	Compound	Retention Time	Mass*	MRM transition	Cone V.	Coll. Energy
1	iP	8.17	204.1	69.1	40	20
				136.2‡	40	10
2	[2H2]iP	8.14	210.1	75.08	40	20
				137.1‡	40	10
3	tZ	2.84	220.3	136.25‡	40	15
				148.25	40	15
				202.3	40	10
4	[2H3]tZ	2.80	225.3	137.25‡	40	15
				148.25	40	15
				207.25	40	10
5	cZ	3.34	220.3	136.25‡	40	15
				148.25	40	15
				202.3	40	10
6	DHZ	3.12	222.3	136.25‡	50	15
				148.2	50	20
				204.2	50	15
7	[2H3]DHZ	3.08	222.3	136.25‡	50	15
				149.2	50	20
8	tZR	5.48	352.1	136.2‡	40	15
				148.2	40	28
				220.2	40	5
9	[2H5]tZR	5.42	357.1	137.2‡	40	15
				148.2	40	28
				225.2	40	10
10	cZR	6.17	352.1	136.2‡	40	15
				148.2	40	28
				220.2	40	5
11	iPR	11.84	336.2	136.2‡	50	15
				148.2	50	15
				204.2	50	10
12	[2H6]iPR	11.82	342.3	137.2‡	50	15
				148.2	50	15
				210.2	50	10

*Mass in positive (+) ion mode. ‡transition used for quantification

Supplemental Table S3. AIC scores for various GAM models of NIN expression. Row names indicate which factors were included in the model. Lower AIC score indicates better model fit.

	df	AIC
Intercept	2	536.151
NO_3^-	3	536.461
LCO	3	465.644
NO_3^- + LCO	9.848	411.748

Supplemental Table S4. Linear model coefficients with 95% confidence intervals and p-values. Modeling the relationship between concentration of six different cytokinins, NO_3^- concentration and LCO application. Model specification: Cytokinin ~ NO_3^- + LCO + NO_3^- : LCO.

	**cZ**	**cZR**	**iP**	**iPR**	**tZ**	**tZR**
NO_3^-	0.001<br>(-0.004, 0.007)<br>p = 0.644	0.007<br>(-0.014, 0.028)<br>p = 0.529	-0.001<br>(-0.005, -0.002)<br>p = 0.474	-0.004<br>(-0.006, 0.014)<br>p = 0.482	-0.001<br>(-0.003, -0.005)<br>p = 0.664	-0.001<br>(-0.002, -0.005)<br>p = 0.422
LCO	-0.016<br>(-0.068, 0.037)<br>p = 0.555	-0.067<br>(-0.273, 0.138)<br>p = 0.523	0.146<br>(0.108, 0.184)<br>$p = 0.000^{***}$	0.380<br>(0.283, 0.477)<br>$p = 0.000^{***}$	0.239<br>(0.198, 0.281)<br>$p = 0.000^{***}$	0.187<br>(0.152, 0.221)<br>$p = 0.000^{***}$
NO_3^- : LCO	0.002<br>(-0.006, 0.010)<br>p = 0.589	0.010<br>(-0.021, 0.040)<br>p = 0.531	-0.005<br>(-0.010, 0.001)<br>p = 0.124	-0.011<br>(-0.025, 0.004)<br>p = 0.157	-0.013<br>(-0.019, -0.007)<br>$p = 0.0001^{***}$	-0.006<br>(-0.012, 0.001)<br>$p = 0.018^{**}$
Intercept	0.154<br>(0.117, 0.191)<br>$p = 0.000^{***}$	0.689<br>(0.544, 0.834)<br>$p = 0.000^{***}$	0.163<br>(0.136, 0.190)<br>$p = 0.000^{***}$	0.286<br>(0.218, 0.355)<br>$p = 0.000^{***}$	0.016<br>(-0.013, 0.045)<br>p = 0.281	0.047<br>(0.022, 0.071)<br>$p = 0.0004^{***}$
Observations	99	99	99	99	99	99
note:						$^{*}p < 0.1$; $^{**}p < 0.05$; $^{***}p < 0.01$
